# Methamphetamine Preconditioning Alters Midbrain Transcriptional Responses to Methamphetamine-Induced Injury in the Rat Striatum

**DOI:** 10.1371/journal.pone.0007812

**Published:** 2009-11-12

**Authors:** Jean Lud Cadet, Michael T. McCoy, Ning Sheng Cai, Irina N. Krasnova, Bruce Ladenheim, Genevieve Beauvais, Natascha Wilson, William Wood, Kevin G. Becker, Amber B. Hodges

**Affiliations:** 1 Molecular Neuropsychiatry Research Branch, DHHS/NIH/NIDA Intramural Research Program, Baltimore, Maryland, United States of America; 2 Gene Expression and Genomics Unit, NIH/NIA Intramural Research Program, Baltimore, Maryland, United States of America; 3 Department of Psychology, Morgan State University, Baltimore, Maryland, United States of America; University of Nebraska, United States of America

## Abstract

Methamphetamine (METH) is an illicit drug which is neurotoxic to the mammalian brain. Numerous studies have revealed significant decreases in dopamine and serotonin levels in the brains of animals exposed to moderate-to-large METH doses given within short intervals of time. In contrast, repeated injections of small nontoxic doses of the drug followed by a challenge with toxic METH doses afford significant protection against monoamine depletion. The present study was undertaken to test the possibility that repeated injections of the drug might be accompanied by transcriptional changes involved in rendering the nigrostriatal dopaminergic system refractory to METH toxicity. Our results confirm that METH preconditioning can provide significant protection against METH-induced striatal dopamine depletion. In addition, the presence and absence of METH preconditioning were associated with substantial differences in the identity of the genes whose expression was affected by a toxic METH challenge. Quantitative PCR confirmed METH-induced changes in genes of interest and identified additional genes that were differentially impacted by the toxic METH challenge in the presence of METH preconditioning. These genes include small heat shock 27 kD 27 protein 2 (HspB2), thyrotropin-releasing hormone (TRH), brain derived neurotrophic factor (BDNF), c-fos, and some encoding antioxidant proteins including CuZn superoxide dismutase (CuZnSOD), glutathione peroxidase (GPx)-1, and heme oxygenase-1 (Hmox-1). These observations are consistent, in part, with the transcriptional alterations reported in models of lethal ischemic injuries which are preceded by ischemic or pharmacological preconditioning. Our findings suggest that multiple molecular pathways might work in tandem to protect the nigrostriatal dopaminergic pathway against the deleterious effects of the toxic psychostimulant. Further analysis of the molecular and cellular pathways regulated by these genes should help to provide some insight into the neuroadaptive potentials of the brain when repeatedly exposed to drugs of abuse.

## Introduction

METH (also nicknamed crank, crystal, speed) is an illicit drug whose abuse prevalence has reached greater proportion than the combined use of heroin and cocaine in the world. The clinical history of METH abuse is characterized by the user initially taking small doses of the drug followed by consumption of progressively larger doses of the psychostimulant [1], [2]. Patients who take these large doses often suffer from a number of psychiatric disorders which include paranoia, psychosis, withdrawal-associated depression, and even suicidal ideations and/or completed suicides [3]. Neuropsychological tests have also revealed significant cognitive deficits in a majority of METH addicts [3], [4]. Evidence for METH-induced structural changes in humans has also accumulated [5], [6]. Several studies have documented decreases in dopamine [7] and of serotonin (5-HT) [8] transporters in various regions of the brain. Although some of these neuropathological changes have been replicated in animal models, their role in the clinical course of METH abuse disorders remains to be clarified [2], [3], [9].

Studies conducted in the 1970's were the first ones to document significant decreases in the levels in DA in the brain of nonhuman primates that had been exposed to repeated injections of METH and sacrificed two weeks after cessation of drug exposure [10]. Subsequent studies in rodents replicated these observations and revealed that METH could cause substantial decreases in DA, 5-HT, and other markers of the integrity of monoaminergic systems in various brain regions [11]–[14]. The majority of these publications used models where moderate to large doses of METH were injected within short intervals of time and/or during single-day binges [2], [15]. Because single-day METH binges are more representative of accidental overdoses by inexperienced users, several groups have experimented with injecting increasing METH doses over several days prior to challenging the animals with large toxic doses of the drug [12], [16], [17]. Although these patterns of drug administration, which we recently entitled METH preconditioning [18], provide substantial attenuation of the toxic effects on monoaminergic systems, the involved neuroprotective mechanisms have remained mysterious.

Several groups of investigators have suggested that METH pretreatment might cause inhibition of METH-induced changes in body temperature, vesicular DA uptake, free radical production, and microglial activation [16], [19], [20] since these are involved in the acute toxic effects of the drug [2]. Nevertheless, much remains to be done in order to identify the molecular pathways involved in the neuroprotection mediated by METH preconditioning. Because the protective effects of METH preconditioning might be related to transcriptional changes similar to those observed in other models of brain preconditioning [21]–[27], the present study was conducted to examine if METH preconditioning might be associated with differential gene expression in the rat midbrain area that encompasses the substantia nigra (SN), a region whose neuronal cell bodies send dopaminergic axons into the rat striatum, a brain region which is injured by toxic METH doses [2]. Identification of these genes might help to decipher molecular mechanisms of protection against METH-induced injuries. These genes might also provide a more systematic rationale for the development of better therapeutic approaches against METH addiction and toxicity.

## Results

### Effects of METH on monoamine levels in the rat brain

**Figure 1:**
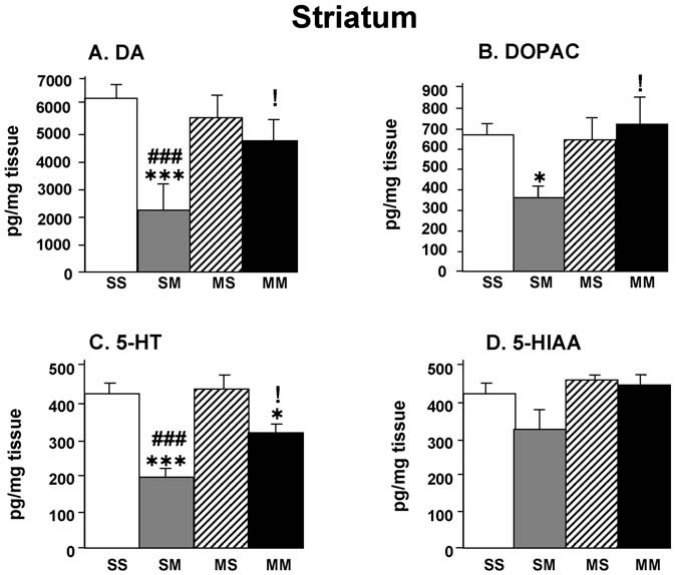
METH preconditioning causes protection against METH-induced depletion of monoamines in the rat striatum. The animals were injected as described in Table S1 and euthanized at 24 hours after the last injection of the saline or METH challenges. Values are expressed as means ± SEM (n = 6-* animals per group). Keys to statistics: *, **, *** = p<0.05, 0.01, 0.001, respectively, in comparison to the SS group; #, ##, ### = p<0.05, 0.01, 0.001, respectively, in comparison to the MS group; !, !! = p<0.05, 0.01, respectively, in comparison to the SM group.

Fig. 1 shows the effects of METH on the levels of DA, DOPAC, 5-HT, and 5-HIAA in the striatum of the animals treated with METH as described in Table S1 provided as supplemental data. Briefly, animals were injected with saline or increasing doses of METH and then challenged 72 hours later with saline or a toxic dose of METH (5 mg/kg×8 injections, given one hour apart). This paradigm resulted in four groups of animals: saline/saline (SS), saline/METH (SM), METH/saline (MS), and METH/METH (MM) [12], [18]. METH preconditioning alone (MS group) did not cause any significant changes in monoamine levels in comparison to saline control group (SS group). The METH challenge caused significant decreases (−63%) in DA levels in the striatum of rats pretreated with saline (SM group) (Fig. 1A). However, the METH challenge caused no significant decreases in DA levels in the METH-preconditioned (MM) group in comparison to the SS or the MS group. In addition, DA levels in the MM group were significantly higher than those measured in the SM group (Fig. 1A). METH challenge also caused significant decreases in DOPAC (−44%) (Fig. 1B) but not in HVA (data not shown) levels in the SM group. Pretreatment with METH provided complete protection against the toxic effects of the METH challenge on DOPAC levels (compare MM group to SM group) (Fig. 1B).

The acute METH challenge also caused substantial decreases in striatal 5-HT concentrations in the saline- (−53%) and METH-pretreated (−24%) rats (Fig. 1C). METH pretreatment provided some degree of protection against reductions in 5-HT levels in drug-challenged animals (compare MM to SM group). 5-HIAA levels showed non-significant changes in the SM group (Fig. 1D). These small decreases were prevented by METH preconditioning (compare SM to MM group) (Fig. 1D).

**Figure 2:**
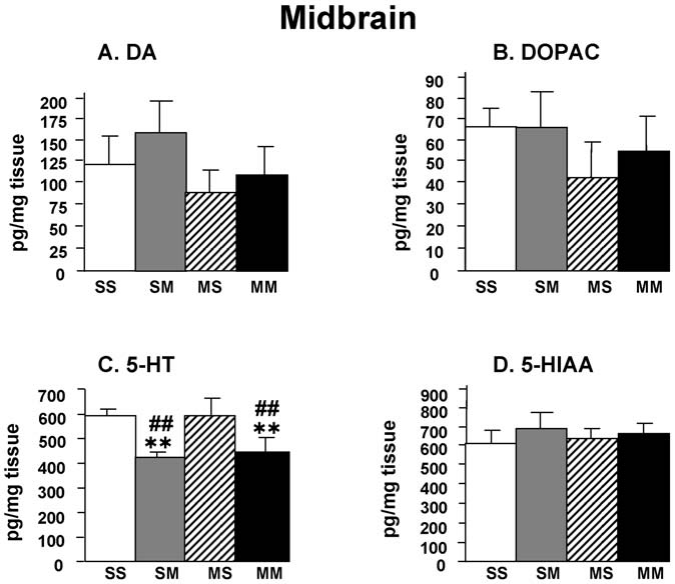
The METH challenge caused serotonin depletion in the ventral midbrain of the rat. The animals were pretreated and challenged with METH or saline as shown in Table S1 and euthanized at 24 hours after the last injection. Keys to statistics are shown in legend to Fig. 1.

Figure 2 shows the effects of METH on the concentrations of DA, DOPAC, 5-HT, and 5-HIAA in a region of the ventral midbrain which encompasses the substantia nigra (SN) and the ventral tegmental area (VTA). Neither METH preconditioning alone nor the METH challenge caused any significant changes in the levels of DA (Fig. 2A) or DOPAC (Fig. 2B) in the SN/VTA area. In contrast, METH injections induced significant decreases in 5-HT levels in both saline- and in METH-pretreated rats (Fig. 2C). However, METH did not cause any changes in 5-HIAA levels in any of the two pretreatment groups (Fig. 2D).

### Identification of genes regulated by METH preconditioning and by METH challenges in the ventral midbrain area

**Figure 3:**
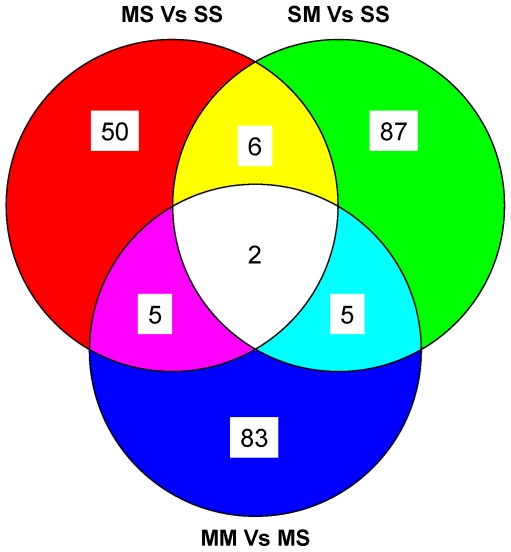
METH preconditioning reconditions midbrain transcriptional responses to METH binge challenges. The Venn diagram shows the overlap of genes identified in the three comparisons. The animals were injected and killed as described above. RNA was extracted from midbrain tissues from the side contralateral to the one used to measure monoamines. Microarray experiments were performed as described in the method section. Genes were identified as significantly changed if they show greater than ±1.7-fold changes at p<0.05.

Microarray analysis has become an important tool in toxicological research because it allows investigators to obtain a better panoramic view of drug-induced transcriptional changes after exposure to pharmacological agents and toxins [28], [29]. In order to assess transcriptional effects of toxic doses of METH in the ventral midbrain of rats pretreated with saline or METH, we used Illumina RatRef-12 Expression BeadChips arrays that contain 22, 523 probes (Illumina Inc., San Diego, CA). The complete raw microarray data have been submitted to the NCBI GEO database: Accession number GSE17665. The Venn diagram in Figure 3 shows the results of 3 comparisons between the four groups of rats: MS vs SS, SM vs SS, and MM vs MS. To be identified as changed, the genes had to meet the following criteria: 1.7-fold changes at p<0.05. A total of 238 showed differential expression in the comparisons that included METH preconditioning alone and toxic METH challenges in the presence or absence of METH preconditioning (see Tables 1–3 for lists of the genes). The METH preconditioning alone caused changes in the expression of 63 genes, with 20 being upregulated and 43 being downregulated (Table 1). These genes fall within classes of cell differentiation, epigenetic control, neurotransmission/signal transduction, and transcription regulation. Interestingly, several synaptic vesicle proteins including synaptogyrin 4, synaptotagmin II and synaptophysin-like 2 [30] are significantly downregulated after chronic administration of METH. The toxic METH challenge caused changes in a total of 100 genes in the absence of METH preconditioning (SM group), with 50 being upregulated and 50 being downregulated (Table 2). These fall within classes of genes involved in metabolism, neurotransmission/signal transduction, proteolysis, responses to various physiological stresses, and transcription control. As expected, the changes in gene expression in animals euthanized 24 hours after the last injection of METH are different from those observed in animals sacrificed at 2 or 4 hours after METH injections that identified changes in immediate early genes (IEGs), several transcription factors, and in genes involved in endoplasmic reticulum stress [31]–[33]. The toxic METH challenge caused differential expression in 95 genes in the METH preconditioned group (MM), with 70 being upregulated and 25 being downregulated (Table 3). These transcripts represent classes of genes that are involved in the control of epigenetic modifications including histone H2ao, neurotransmission/signal transduction such as thyrotropin releasing hormone (TRH), and stress responses including heat shock protein 27kd protein 2 (Hspb2). Surprisingly, only two genes, namely olfactory receptor 1143 and ribosomal protein L36a, were common among the three sets of comparisons (Fig. 3). They were both downregulated in the MS/SS and SM/SS comparisons but upregulated in the MM/MS comparison (see Tables 1–3).

**Table 1:** Effects of METH preconditioning alone on gene expression in the ventral midbrain.

Genbank	Symbol	Gene Name	MS/SS
	***Epigenetics***		
XM_344599	**Hist1h2ao**	histone 1, H2ao	**−2.04**
NM_001106371	**Hells**	helicase, lymphoid specific	**−40.47**
	***Metabolism and Catabolism***		
XM_230581	**Acoxl**	acyl-Coenzyme A oxidase-like	**17.92**
NM_001025402	**Umps**	uridine monophosphate synthetase	**−2.32**
NM_031598	**Pla2g2a**	phospholipase A2, group IIA (platelets, synovial fluid)	**−64.48**
	***Neurotransmission/Signal Transduction***		
NM_001001023	**Olr94**	olfactory receptor 94	**16.34**
NM_001000409	**Olr855**	olfactory receptor 855	**1.81**
NM_001007557	**Emr1**	EGF-like module containing, mucin-like, hormone receptor-like 1	**−1.70**
NM_001000129	**Olr62**	olfactory receptor 62	**−1.84**
NM_012665	**Syt2**	synaptotagmin II	**−1.84**
NM_012627	**Pkib**	protein kinase (cAMP-dependent, catalytic) inhibitor beta	**−1.94**
NM_001025644	**Syngr4**	synaptogyrin 4	**−2.01**
NM_001000915	**Olr790**	olfactory receptor 790	**−2.34**
XM_001068241	**Eda2r**	ectodysplasin A2 receptor	**−2.72**
NM_001109144	**Pth2**	parathyroid hormone 2	**−4.15**
NM_001009487	**Ly49s4**	Ly49 stimulatory receptor 4	**−7.38**
NM_080773	**Chrm1**	cholinergic receptor, muscarinic 1	**−11.13**
NM_001107800	**Stk4**	serine/threonine kinase 4	**−16.69**
NM_001107188	**Rasal2**	RAS protein activator like 2	**−17.36**
NM_001001017	**Olr1143**	olfactory receptor 1143	**−18.65**
NM_001005384	**Osmr**	oncostatin M receptor	**−22.22**
NM_001108563	**Sypl2**	synaptophysin-like 2	**−22.29**
NM_001081444	**Pik3r6**	phosphoinositide-3-kinase, regulatory subunit 6	**−22.94**
	***Transcription/Transcription Factors/Nucleotide Binding***		
NM_052802	**Kap**	kidney androgen regulated protein	**29.67**
XM_342933	**Gpatch3**	G patch domain containing 3	**15.80**
NM_022857	**N5**	DNA binding protein N5	**−1.80**
NM_001106375	**Papss2**	3′-phosphoadenosine 5′-phosphosulfate synthase 2	**−1.85**
NM_001108953	**Zbtb6**	zinc finger and BTB domain containing 6	**−2.37**
NM_001137626	**E2f3**	E2F transcription factor 3	**−25.45**
NM_001107170	**Tcfcp2l1**	transcription factor CP2-like 1	**−26.76**
NM_001105863	**Thap7**	THAP domain containing 7	**−34.62**

The data in this table were generated from the comparisons between the METH preconditioning alone (MS group) and saline control group (SS group) of animals euthanized at 24 h. To be identified as changed, the genes had to meet the criteria: greater or less than 1.7-fold and p<0.05. The values represent fold changes between the specified groups (n = 4 per group). The genes are listed in descending order according to the METH-induced fold changes within their specific functional classification.

**Table 2 pone-0007812-t002:** METH challenge-induced gene expression in the absence of METH preconditioning.

Genbank	Symbol	Gene Name	SM/SS
	***Epigenetics***		
NM_001108060	**Rcor1**	REST/NRSE corepressor 1	**29.50**
	***Metabolism and Catabolism***		
XM_001075890	**Foxred2**	FAD-dependent oxidoreductase domain containing 2	**4.76**
XM_001074061	**Mthfr**	5,10-methylenetetrahydrofolate reductase	**3.08**
NM_053962	**Sds**	serine dehydratase	**3.05**
NM_001004077	**Gk2**	glycerol kinase 2	**2.23**
NM_001109022	**Inmt**	indolethylamine N-methyltransferase	**2.00**
NM_053896	**Aldh1a2**	aldehyde dehydrogenase 1 family, member A2	**1.79**
NM_031834	**Sult1a1**	sulfotransferase family, cytosolic, 1A, phenol-preferring, member 1	**1.78**
NM_001105899	**Liph**	lipase, member H	**−1.72**
NM_031010	**Alox15**	arachidonate 15-lipoxygenase	**−2.68**
NM_012496	**Aldob**	aldolase B	**−3.40**
XM_001068364	**Akr1c12**	aldo-keto reductase family 1, member C12	**−31.03**
	***Neurotransmission/Signal Transduction***		
NM_001106879	**Efhb**	EF hand domain family, member B	**55.87**
NM_001107909	**Map3k6**	mitogen-activated protein kinase kinase kinase 6	**40.00**
NM_001044250	**Stat6**	signal transducer and activator of transcription 6	**21.74**
NM_001008932	**V1rg17**	vomeronasal 1 receptor, G17	**21.65**
NM_031649	**Klrg1**	killer cell lectin-like receptor subfamily G, member 1	**17.70**
NM_001107726	**Rrh**	retinal pigment epithelium derived rhodopsin homolog	**16.92**
XM_001066511	**Pdzd3**	PDZ domain containing 3	**7.41**
NM_012835	**Cort**	cortistatin	**4.41**
NM_001108975	**Ptch1**	patched homolog 1	**2.37**
NM_138505	**Adra2b**	adrenergic, alpha-2B-, receptor	**2.24**
XM_001080694	**Ccdc155**	coiled-coil domain containing 155	**1.96**
NM_057115	**Ptpn12**	protein tyrosine phosphatase, non-receptor type 12	**1.75**
NM_001033064	**Kazald1**	Kazal-type serine peptidase inhibitor domain 1	**−1.73**
NM_001000782	**Olr1414**	olfactory receptor 1414	**−1.74**
NM_031766	**Cpz**	carboxypeptidase Z	**−1.75**
XM_232745	**Sfn**	stratifin	**−1.78**
NM_001008513	**Ccl21b**	chemokine (C-C motif) ligand 21b (serine)	**−1.93**
NM_001106894	**Gpr110**	G protein-coupled receptor 110	**−1.99**
NM_012770	**Gucy1b2**	guanylate cyclase 1, soluble, beta 2	**−2.03**
NM_001106123	**Mrc1**	mannose receptor, C type 1	**−2.11**
NM_001000268	**Olr1673**	olfactory receptor 1673	**−2.12**
NM_001000132	**Olr49**	olfactory receptor 49	**−2.40**
NM_022202	**Grm8**	glutamate receptor, metabotropic 8	**−3.06**
NM_181373	**Grik3**	glutamate receptor, ionotropic, kainate 3	**−6.13**
NM_001107625	**Plekhk1**	pleckstrin homology domain containing, family K member 1 Rtkn2 rhotekin 2	**−12.91**
NM_001000884	**Olr1117**	olfactory receptor 1117	**−17.92**
NM_012609	**Nf1**	neurofibromin 1	**−24.98**
NM_001001017	**Olr1143**	olfactory receptor 1143	**−41.75**
NM_001000151	**Olr113**	olfactory receptor 113	**−74.45**
	***Stress Responses***		
NM_057194	**Plscr1**	phospholipid scramblase 1	**5.43**
NM_001109577	**Derl3**	Der1-like domain family, member 3	**2.27**
NM_001007729	**Pf4**	platelet factor 4	**−1.76**
NM_019335	**Eif2ak2**	eukaryotic translation initiation factor 2-alpha kinase 2	**−2.15**
NM_133624	**Gbp2**	guanylate nucleotide binding protein 2	**−2.39**
NM_182952	**Cxcl11**	chemokine (C-X-C motif) ligand 11	**−4.90**
NM_012725	**Klkb1**	kallikrein B, plasma 1	**−103.20**
	***Transcription/Transcription Factors/Nucleotide Binding***		
XM_220520	**Rai1**	retinoic acid induced 1	**33.78**
NM_145767	**Prrxl1**	paired related homeobox protein-like 1	**2.69**
NM_017058	**Vdr**	vitamin D (1,25- dihydroxyvitamin D3) receptor	**2.15**
NM_001104612	**Jrk**	jerky homolog (mouse)	**1.98**
XM_216941	**Matn2**	matrilin 2	**1.90**
NM_001033691	**Irf7**	interferon regulatory factor 7	**−1.88**
XM_341433	**Ccdc111**	coiled-coil domain containing 111	**−1.95**
NM_001107281	**Klf12**	Kruppel-like factor 12	**−2.96**
NM_053520	**Elf1**	E74-like factor 1	**−16.05**

The data in this table were generated from the comparisons between the saline-pretreated challenged with METH (SM group) and saline control group (SS group) of animals euthanized at 24 h. To be identified as changed, the genes had to meet the criteria: greater or less than 1.7-fold and p<0.05. The values represent fold changes between the specified groups (n = 4). The genes are listed in descending order according to the METH-induced fold changes within their specific functional classification.

**Table 3:** METH challenge-induced gene expression in the presence of METH preconditioning.

Genbank	Symbol	Gene Name	MM/MS
	***Epigenetics***		
XM_001052969	**Tert**	telomerase reverse transcriptase	**15.60**
XM_344599	**Hist1h2ao**	histone 1, H2ao	**1.76**
	***Metabolism and Catabolism***		
NM_001024321	**Hyal5**	hyaluronoglucosaminidase 5	**21.41**
NM_001012080	**Hfe2**	hemochromatosis type 2 (juvenile) (human homolog)	**3.40**
NM_001031656	**Serinc2**	serine incorporator 2	**3.09**
NM_001025402	**Umps**	uridine monophosphate synthetase	**2.92**
NM_022926	**Galnt7**	UDP-N-acetyl-alpha-D-galactosamine:polypeptide N-acetylgalactosaminyltransferase 7 (GalNAc-T7)	**2.06**
NM_173308	**Fut11**	fucosyltransferase 11	**2.03**
NM_173303	**Cox6c1**	cytochrome c oxidase subunit VIc-1	**1.81**
XM_227543	**Man1a2**	mannosidase, alpha, class 1A, member 2	**1.77**
NM_031582	**Aoc3**	amine oxidase, copper containing 3 (vascular adhesion protein 1)	**−1.99**
XM_230581	**Acoxl**	acyl-Coenzyme A oxidase-like	**−17.93**
	***Neurotransmission/Signal Transduction***		
XM_345342	**C5**	complement component 5	**48.31**
NM_172328	**Tac4**	tachykinin 4, Preprotachykinin C	**35.59**
NM_017123	**Areg**	amphiregulin	**33.33**
NM_001001017	**Olr1143**	olfactory receptor 1143	**16.34**
NM_019630	**Gip**	gastric inhibitory polypeptide	**16.31**
XM_341088	**Rasal1**	RAS protein activator like 1	**15.24**
NM_001009967	**Pip5k1c**	phosphatidylinositol-4-phosphate 5-kinase, type I, gamma	**13.72**
NM_001001026	**Olr127**	olfactory receptor 127	**13.23**
XM_343640	**Ptprm**	protein tyrosine phosphatase, receptor type, M	**13.16**
XM_343881	**Havcr2**	hepatitis A virus cellular receptor 2	**12.97**
NM_001107909	**Map3k6**	mitogen-activated protein kinase kinase kinase 6	**10.03**
NM_013046	**Trh**	thyrotropin releasing hormone	**4.22**
NM_022714	**Crhr2**	corticotropin releasing hormone receptor 2	**3.02**
NM_058208	**Socs2**	suppressor of cytokine signaling 2	**2.52**
NM_139188	**Otos**	otospiralin	**2.35**
XM_001058249	**Fcrl1**	Fc receptor-like 1	**2.03**
XM_001055537	**Rhbdl2**	rhomboid, veinlet-like 2 (Drosophila)	**1.98**
XM_001075502	**Ms4a11**	membrane-spanning 4-domains, subfamily A, member 11	**1.90**
XM_213380	**Rilp**	Rab interacting lysosomal protein	**1.78**
NM_021684	**Adcy10**	adenylate cyclase 10 (soluble)	**−1.97**
NM_001108321	**Rtp4**	receptor transporter protein 4	**−2.02**
XM_344047	**Olr1571**	olfactory receptor 1571	**−4.34**
NM_133413	**Cysltr2**	cysteinyl leukotriene receptor 2	**−12.54**
NM_001000146	**Olr105**	olfactory receptor 105	**−20.33**
	***Stress Responses***		
NM_212488	**Btnl7**	butyrophilin-like 7	**164.20**
NM_130431	**Hspb2**	heat shock protein 2	**14.03**
XM_574098	**Mtcp1**	mature T-cell proliferation 1	**1.70**
XM_001057564	**Csf3r**	colony stimulating factor 3 receptor (granulocyte) (Csf3r)	**−1.81**
NM_145672	**Cxcl9**	chemokine (C-X-C motif) ligand 9	**−3.58**
	***Transcription/Transcription Factors/Nucleotide Binding***		
XM_224295	**Zc3h13**	zinc finger CCCH type containing 13	**34.01**
NM_207611	**Bhlhb9**	basic helix-loop-helix domain containing, class B, 9	**26.74**
NM_031803	**Gmeb2**	glucocorticoid modulatory element binding protein 2	**25.77**
NM_001109237	**Neurod6**	neurogenic differentiation 6	**2.54**
XM_215728	**Hltf**	helicase-like transcription factor	**2.41**
NM_001037216	**Nxf7**	nuclear RNA export factor 7	**−1.74**
NM_139186	**Mki67ip**	spermatogenesis-related protein	**−1.84**
XM_001058675	**Rorc**	RAR-related orphan receptor C	**−1.93**
XM_221915	**Zfp853**	zinc finger protein 853	**−2.02**
NM_053468	**Rag1**	recombination activating gene 1	**−2.12**
NM_001033691	**Irf7**	interferon regulatory factor 7	**−2.74**
NM_001025729	**Zbed3**	zinc finger, BED domain containing 3	**−6.25**

The data in this table were generated from the comparisons between the METH preconditioning treated with METH (MM group) and METH preconditioning alone (MS group) of animals euthanized at 24 h. To be identified as changed, the genes had to meet the criteria: greater or less than 1.7-fold and p<0.05. The values represent fold changes between the specified groups (n = 4). The genes are listed in descending order according to the METH-induced fold changes within their specific functional classification.

One interesting observation among the response profiles occurs between the saline- and METH-pretreated rats after injections of toxic doses of METH. The Venn diagram showed that only 7 genes overlapped between these two sets of comparisons (Fig. 3). These include interferon regulatory factor 7 (Irf7), matrix metallopeptidase 14 (Mmp14), mitogen-activated protein kinase kinase kinase 6 (Map3k6), olfactory receptor 1143 (Olr1143), parvin beta (parvinb), and ribosomal protein L36a (Rpl36a), The observation of very few overlapping genes suggests that transcriptional responses to a METH challenge in the presence and absence of METH preconditioning are very dissociable (compare the list of genes in Tables 2 and 3). In fact, although some of them fell within similar classifications, as noted above, there were marked differences in the identity of the METH-regulated genes in the absence and presence of METH preconditioning. For example, there was no overlap in the genes listed under classes of epigenetics, metabolism and catabolism, responses to stress, or cellular transport. Moreover, of the overlapped genes, Irf7, Mmp14, and Parvb were downregulated after the METH challenge in both the absence and presence of METH preconditioning, Map3k6 was upregulated in both cases, whereas Olr1143 and Rpl36a were downregulated or upregulated in the respective absence or presence of METH pretreatment. One possibility for these differences is that the METH challenge caused increased expression of the repressor element silencing transcription factor/neuronal restrictive silencer factor (REST/NRSF) [34], [35] corepressor 1 (Rcort1) in the absence of METH preconditioning (see Table 3). The REST corepressor acts together with REST to silence the expression of many genes [35] which represent various functional groups including ion channels, metabolism, neurotransmitter receptors, and intracellular signaling [36]. Thus, METH-induced upregulation of this co-repressor might be responsible, in part, for the larger number of genes that are downregulated in the SM in contrast to the MM group (compare Table 2 to Table 3).

### Quantitative PCR

**Figure 4:**
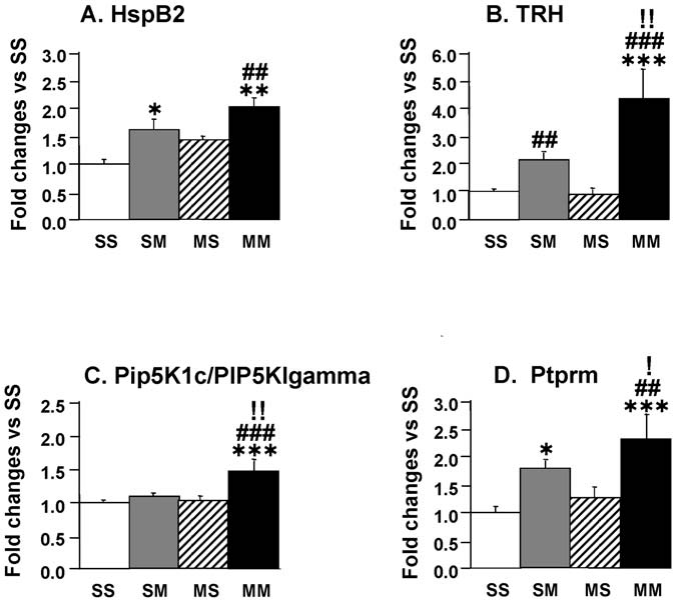
Quantitative PCR validates METH challenge-induced changes in gene expression in the METH-preconditioned group. Data were obtained from RNA obtained from 5–6 animals per group and measured individually. The mRNA levels were normalized to 18S rRNA levels. The values represent means + SEM in comparison to the saline-pretreated challenged with saline (SS group). METH caused substantial increases in (A) HspB2 in the MM group, (B) TRH in the SM and MM groups, (C) Pip5k1c in the MM group, and (D) Ptprm in both the SM and MM groups. Keys to statistics: *, **, *** = p<0.05, 0.01, 0.001, respectively, in comparison to the SS group; #, ##, ### = p<0.05, 0.01, 0.001, respectively, in comparison to the MS group; !, !!, !!! = p<0.05, 0.01, 0.001 respectively, in comparison to the SM group.

We used quantitative PCR to validate the expression of some of the genes identified by the microarray analyses using RNA from individual animals from the four groups. The primers are listed in Table 4. We first confirmed the METH-induced changes in the expression of HspB2 [37], [38] which is a member of the family of small heat shock proteins (sHSPs) that have been shown to exert significant protective effects in models of neurodegeneration [39], [40]. As seen in Fig. 4A, the METH challenge caused significant changes in HspB2 expression in both the presence and absence of METH preconditioning, with the increases in the METH preconditioned group being of greater magnitude. HspB2 is localized in the mitochondria and protects cells against heat-mediated cell demise [41]. Experiments using knockout mice have shown that HspB2 can protect against ischemia/reperfusion-induced injuries in the heart [42], suggesting that the METH-induced changes in HspB2 might participate in preventing retrograde degeneration of the nigrostriatal dopaminergic system in rodents after METH-induced destruction of striatal DA terminals.

**Table 4:** List of Primers.

Primer Name	Primer Up	Primer Down
HspB2	CTG CCG AGT ACG AAT TTG CC	CTC TGG CTA TCT CTT CCT CTT
TRH	GGA CAA GTA TTC ATG GGC	CTC TTG GTG ACA TCA GAC
Pip5K1c	GCC TCT GAT GAG GAA GAT GC	AGT TAT GTG TCG CTC TCG CC
Ptprm	TCA TCG ACC CAA CCA TTA T	CCA GTA TTT GCA GCA TTT C
c-fos	GGG CAA AGT AGA GCA G	CTC TTT CAG TAG ATT GGC A
Fra1	TGT GCC AAG CAT CAA C	CCA ACT TGT CGG TCT C
Fra2	CTG TGT GCA AAA TCA GT	AGC AAT GCT AAT GGG C
c-Jun	TTG CCC CAA CAG ATC C	GCT GCG TTA GCA TGA G
JunB	CAC GAC TAC AAA CTC C	CGT GGT TCA TCT TCT G
JunD	GTG TGT TTC CTT GTG TTG	TTT GGC GTA ACG AAG AC
BDNF	TGA TGC TCA GCA GTC AA	CAC TCG CTA ATA CTG TCA C
GDNF	GGA CTC TAA GAT GAA GTT ATG G	ATC AAA CTG GTC AGG ATA AT
CuZnSOD	AAT ACA CAA GGC TGT ACC	GAG ATC ACA CGA TCT TCA A
MnSOD	AAC TGG GAG AAT GTT AGC	TGG ATA GGC ATC AAT GAA GAT TA
GPx-1	TGT TTG AGA AGT GCG AG	TCC AGG AAA TGT CGT TG
Hmox-1	GTA CCA TAT CTA CAC GGC	GGA GAC GCT TTA CAT AGT
18s	GCG CAA ATT ACC CAC T	ATC CAA CTA CGA GCT T

We also sought to confirm the METH-induced changes in the expression of thyrotropin-releasing hormone (TRH) observed in the microarray data because TRH is widely distributed in the rat brain [43] and interacts with dopaminergic systems in the brain [44]. Our results confirmed that the METH challenges caused substantial increases in TRH expression in the presence of METH preconditioning (Fig. 4B). There were also METH-induced changes in the saline-pretreated rats, increases that were of smaller magnitude than those observed in the presence of METH preconditioning. The small increases observed in the SM group are consistent with increases in TRH concentrations previously reported in the brains of rats that had received doses of the neurotoxin, 6-hydroxydopamine (6-OHDA), which depletes DA in the brain [45] since the SM group also experiences significant decreases in striatal DA levels (see Fig. 1A). The fact that METH-challenged METH-preconditioned animals, which were protected against striatal DA depletion (MM group in Fig. 1A), showed greater increases in TRH expression than the saline-pretreated METH-challenged rats suggests that the changes in the TRH transcript in the former group might be involved not only in protecting the cell bodies located in the SN/VTA area but also in protecting striatal dopaminergic terminals against METH-induced DA depletion.

As shown in Fig. 4C, we were also able to confirm the METH-induced increases in Pip5k1c, also called PIP5KIgamma [46]. The METH challenge caused increases only in the METH preconditioned state. Pip5k1c is a major synaptic type I phosphatidylinositol 4-phosphate (PtdIns(4)P) 5-kinase (PIP5K) that phosphorylates phosphatidylinositol-4-phosphate to generate phosphatidylinositol 4, 5-bisphosphate (PIP2), a lipid molecule that has been implicated in an array of cellular functions which include signal transduction, cytoskeletal organization, regulated exocytosis and clathrin-mediated endocytosis [47]–[50]. Because PIP2 is also involved in the mediation of gene expression and cell survival [51], [52], the present results suggest that METH preconditioning might have induced changes in lipid signaling, which might participate in the alterations of METH-induced transcriptional responses of the SN/VTA cell bodies. Because PIP5K function is regulated mostly through protein interactions with Rho and Arf families of small G-proteins [53], it was surprising that METH preconditioning was associated with increased Pip5k1c transcription in response to a toxic METH challenge. Elucidation of the mechanism involved and the role of Pip5k1c in the function of the mesostriatal dopaminergic system in the absence and presence of METH preconditioning will have to await future studies. This is an important issue because PIP5KIgamma is the major PIP kinase identified at synapses [54] and because it has recently been shown to be required for neuronal development [55].

We also confirmed METH-induced increases in Ptprm in the presence of METH preconditioning (Fig. 4D). We also found that the METH challenge caused smaller increases in the saline-pretreated group. Ptprm is a member of the family of tyrosine phosphatases which are involved in tyrosine phosphorylation/dephosphorylation events that are controlled by protein tyrosine kinases (PTKs) and protein tyrosine phosphatases (PTPs) [56]–[58]. This process is critical to the regulation of several cellular functions including cell proliferation and differentiation, metabolism, and gene transcription [58]. Ptprm mediates aggregation through homophilic binding [59] and associates with cadherins [60] which are a large family of cell-cell adhesion molecules that bind actin and intermediate filaments to the plasma membrane and play significant roles in synaptic plasticity [61]. Thus, increased Ptprm expression in the presence of METH preconditioning might constitute one component of molecular events involved in long-term neuroadaptations to repeated METH injections. This idea is consistent with reports of psychostimulant-induced structural plasticity in animals exposed to psychostimulants [62].

**Figure 5:**
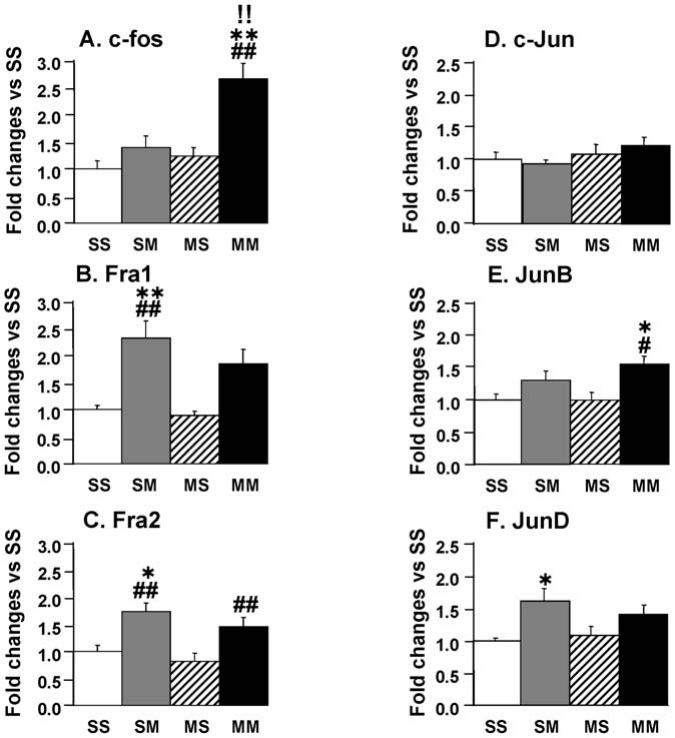
The METH challenge caused differential responses in the expression of AP1 transcription factors in the presence and absence of METH preconditioning. METH caused substantial increases in (A) c-fos in the MM group, (B) Fra1 in the SM group, and (C) Fra2 in the SM and MM groups. (D) c-Jun expression was not affected in any of the groups whereas (E) JunB showed METH-induced increases in the MM while (F) JunD expression was increased in the SM group. Keys to statistics are as described in Fig. 4.

Microarray analyses may sometimes underestimate changes in gene expression [63]. Therefore, we quantified the expression of some members of the AP1 transcription factors (TFs) which have been implicated in brain preconditioning [64], [65] and are affected in several regions of the rodent brain early after METH administration [31], [32], [66], [67]. We thought it possible that there might be differential expression of these factors in the METH preconditioning model even though they were not identified in the microarray experiments. Fig. 5 shows the effects of the toxic METH challenge in the absence and presence of METH preconditioning. There were significant increases in c-fos expression in the METH-challenged preconditioned group in comparison to all three groups (Fig. 5A). The METH-challenged saline-pretreated group did not show any significant changes in c-fos. Fra1 expression showed significant METH challenge-induced increases in the absence of METH preconditioning whereas the increases in the MM group did not reach statistical significance (Fig. 5B) There were significant increases in Fra2 expression in the SM group in comparison to both the SS and the MS group whereas the increased observed in the MM group were significantly different from the MS group (Fig. 5C). We also measured the expression of c-jun, junB, and junD in the four groups. There were no significant different differences in c-jun expression in any of the groups (Fig. 5D). METH caused significant increases only in the METH preconditioned state (Fig. 5E). JunD expression was affected by METH only in the absence of METH preconditioning (Fig. 5F).

**Figure 6:**
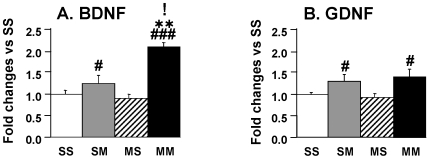
The METH challenge caused increases in BDNF expression in the rat ventral midbrain in the presence of METH preconditioning. The METH challenge caused significant increases in (A) BDNF mRNA in the METH-preconditioned group (MM). The METH-induced changes in (B) GDNF were only significantly different from the values in the MS but not from the other groups. Keys to statistics are as in Fig. 4.

We also measured the expression of brain derived neurotrophic factor (BDNF) which is thought to be a mediator of ischemic preconditioning [64], [65], [68], [69] and of glial cell line-derived neurotrophic factor (GDNF) which is known to exert protective effects against METH-induced toxicity [70]–[72]. Both of these trophic factors are involved in the survival of midbrain dopaminergic neurons [73]–[76]. Fig. 6A shows that the toxic METH challenge caused significant increases in BDNF in the presence of METH preconditioning. The small increase in BDNF in the SM group was not significantly different from the SS group but was different from the MS group. In contrast, there were smaller changes in the expression of GDNF after the METH challenge in the presence and absence of METH preconditioning, with these changes being significant only in comparison to the MS group (Fig. 6B).

**Figure 7:**
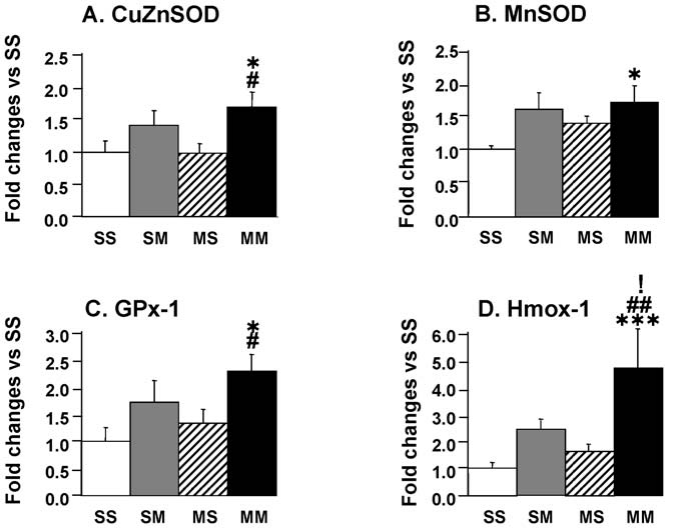
The METH challenge caused changes in the expression of antioxidant transcripts in the ventral midbrain of METH-preconditioned rats. The METH challenge caused significant increases in (A) CuZnSOD, (B) MnSOD, (C) GPx, and (D) Hmox1 in the METH-preconditioned group. Keys to statistics are shown in the legend to Fig. 4.

Finally, we measured the expression of several antioxidant genes that have been proposed as potential mediators of ischemic preconditioning [64], [77], [78] because METH toxicity involves the production of reactive oxygen species [2], [79]. These include the antioxidant enzymes copper zinc superoxide dismutase (CuZnSOD), manganese SOD (MnSOD), and glutathione peroxidase-1 (GPx1). These were chosen because METH-induced toxicity is mediated, in part, by the production of reactive oxygen species (ROS) such as superoxides [80], hydrogen peroxide (H2O2) [81], [82] and hydroxyl radicals (see [2] for a recent review). We wanted to know if these antioxidant genes might also show differential responses to the METH challenge in the presence and absence of METH preconditioning. Fig. 7A shows that the METH challenge caused small but significant increases in CuZnSOD mRNA levels in the MM in comparison to the SS and MS groups. The SM group showed small increases that did not reach significance. The expression of MnSOD was also significantly increased in the MM group (Fig. 7B). The METH-induced changes in the SM group did not reach significance. GPx-1 showed significant increases in the MM group but not in the SM group (Fig. 7C). We also measured the levels of Hmox-1 which is induced by toxic METH doses [33] and which was recently reported to protect against METH toxicity [83]. As can be seen in Fig. 7D, there were significant METH challenge-induced increases in Hmox-1 expression in the presence of METH preconditioning. The increases in the SM did not reach significance due to some individual variability in the response to METH in that group.

## Discussion

The major findings of our study are that a challenge with toxic doses of METH caused marked reductions in the levels of DA and 5-HT in the striatum but significant decreases only in 5-HT concentrations in the VTA/SN of rats. Pretreatment of the animals with progressively higher but nontoxic doses of METH caused complete protection against METH challenge-induced DA depletion but partial protection against 5-HT depletion in the striatum. In contrast, the pretreatment did not afford any protection against METH-induced decreases in 5-HT levels in the ventral midbrain. The observations on the protective effects of METH preconditioning on drug-induced monoamine depletion are consistent with those reported by several groups of investigators (reviewed in [2]). In addition to the biochemical data, microarray analyses revealed that METH preconditioning was associated with METH challenge-induced transcriptional responses that were substantially different from those observed in the absence of drug preconditioning. The transcriptional profile in response to METH preconditioning alone is characterized by significant decreases in the expression of several transcripts (43 out of 63 genes) that are involved in epigenetic phenomena, neurotransmission and signal transduction, and transcriptional regulation (see Table 1). These observations suggest that the latent METH tolerant brain might be characterized by a state of decreased metabolism associated with suppressed neurotransmission because 18 of 21 genes involved in metabolism, neurotransmission and signal transduction were down-regulated by METH preconditioning alone. This idea is consistent with clinical studies that have reported that humans who abuse METH chronically show decreased brain glucose metabolism [84], [85]. Our additional findings that pretreatment with progressively increasing nontoxic amount of METH is associated with substantial alterations in the transcriptional responses to an injurious METH challenge are consistent with observations that brief ischemic events can also change genomic responses to more prolonged ischemic injuries [25], [26], [86]. These results suggest that neuroadaptive molecular changes might serve a fundamental role in the survival of neurons in organisms faced with an array of environmental toxic stressors [87], [88] . In what follows, we discuss the potential protective role of differential changes in gene expression in the model of METH preconditioning.

TRH was originally discovered as a hypothalamic neuropeptide which is involved in the synthesis and release of thyrotropin for the pituitary gland [89]. TRH is widely distributed in the brain [43] and is important in the regulation of energy metabolism via effects on feeding behaviors, locomotor activity and thermogenesis [90], [91]. A number of studies have indicated that TRH and some analogs can provide significant neuroprotection in several models of neurodegeneration. For example, TRH has been shown to improve recovery after traumatic injuries to the cervical spine [92] and the brain [93]. It has also been reported that TRH analogs also provide significant beneficial effects against cerebral ischemia [94], [95]. TRH also provides neuroprotection against N-methyl-D-aspartate (NMDA)-induced cell death in rat hippocampal slices [96]. This discussion is also consistent with reports that TRH can protect against kainate-induced neurotoxicity in rodents [97] and glutamate-induced neuronal cell death [98]. Since ischemic or pharmacological preconditioning provides significant protection in these various models [27], [64], [99], it will be of interest to test whether these preconditioning paradigms also cause increases in TRH expression.

The expression of BDNF, a member of the neurotrophin family of trophic factors that are involved in the developmental regulation of cell survival and differentiation and in the mediation of synaptic plasticity [100], [101], was also affected differentially by the METH challenge in the absence and presence of METH preconditioning. The increases in the BDNF transcript in the METH-preconditioned animals which were then injected with toxic doses of METH suggest that the repeated injections of nontoxic doses of METH might have primed the BDNF promoter, possibly via epigenetic modifications, to such a degree that there were increased BDNF transcription only after exposure to a toxic dose of the drug since there were no changes in the BDNF transcript in the METH preconditioning only group. The idea that the BDNF responses to METH preconditioning might be related to epigenetic changes is consistent with the report that histone deacetylase (HDAC) inhibitors can cause increases in BDNF transcription and protection of dopaminergic neurons against cellular damage [102]. Moreover, the observations that BDNF expression is related to decreases in CpG methylation in the regulatory sequence of the BDNF gene [103] and that developmental BDNF expression in the mouse brain is also correlated with patterns of methylation at CpG sites within the BDNF promoter [104] also support the idea that epigenetic phenomena are very important to the regulation of BDNF expression after METH preconditioning. The possibility exists, nevertheless, that other mechanisms might be involved in BDNF regulation. For example, we found that METH caused differential c-fos expression in a manner that parallels the changes in BDNF expression among the experimental groups. Members of the AP-1 family of transcription factors, especially c-fos, are induced in several models of neuronal injuries [105], [106]. BDNF is often induced in the same models of brain injury [105], [106], with BDNF and c-fos being, oftentimes, co-induced in neurons after excitotoxic damage [105]. Moreover, c-fos mutant knockout mice show altered responses in BDNF expression after injections of the excitotoxin, kainic acid [105], [106]. Also of interest is the demonstration that BDNF can induce c-fos expression in midbrain dopaminergic neurons [107]. Thus, when taken together with our present data, these observations suggest that the METH-induced increases in BDNF expression observed after METH preconditioning might, in part, be secondary to METH-induced changes in c-fos expression or vice versa in such a manner as to form a feedback regulatory loop that serves to provide long-term neuroprotection against METH-induced injuries. The latter suggestion is consistent with our previous observation that METH toxicity is exacerbated in the brains of c-fos knockout mice [108]. This idea is also supported by the report that induction of endogenous BDNF protects midbrain DA neurons against kainate-induced transneuronal degeneration [73]. It is also remarkable that BDNF has been reported to cause upregulation of pre-pro-TRH in the fetal hypothalamus [109], [110]. These observations suggest that BDNF might act through various signaling mechanisms to protect the mesostriatal DA system against the toxic effects of METH since TRH, a neuroprotective hormone [92]–[96], showed large increases in the ventral midbrain of METH-challenged rats in the presence of METH preconditioning.

**Figure 8:**
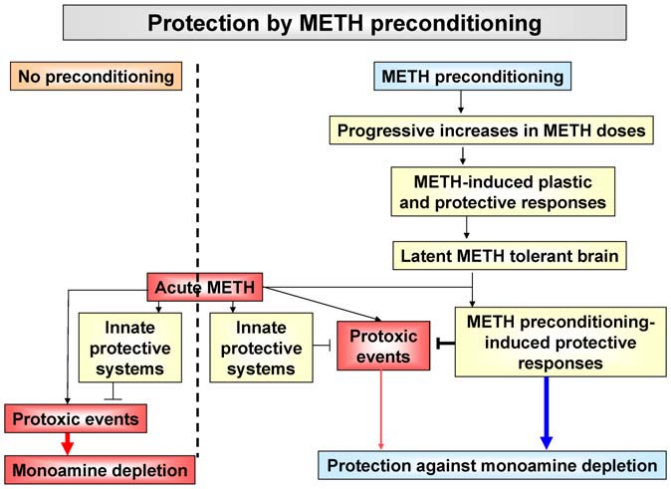
Schematic rendering of potential pathways involved in METH preconditioning-induced protection on METH-induced striatal DA depletion. The METH challenge caused substantial depletion of monoamines in the saline-pretreated animals. Repeated injections of lower nontoxic doses of METH can cause repeated low levels of oxidative stress that are not toxic to cells. Moreover, repeated non-toxic oxidative stress in the striatum and/or the ventral midbrain might trigger molecular mechanisms that generate a state of latent METH refractory brain that provides protection against METH toxicity. The proposed tolerant state might occur through chronic METH-induced free radical-mediated epigenetic changes and subsequent differential genomic responses to toxic doses of the drug.

Recent evidence has accumulated to suggest that some of the protective effects of trophic factors, including BDNF, might be mediated through inhibition of the deleterious effects of reactive oxygen species (ROS) [111]–[113]. ROS including superoxide radicals, hydrogen peroxide, and hydroxyl radicals are reactive molecules that are produced during normal cellular processes [114]. Their overproduction in the brain is thought to negatively impact protein function, to cause lipid peroxidation, damage to nucleic acids and to be involved in neurodegenerative processes [114], [115]. Almost immediately after the description of the toxic effects of METH, it was suggested that METH-induced monoamine depletion might be mediated by reactive species generated during DA metabolism [116]. A role for superoxide radicals was confirmed by the demonstration that METH toxicity was attenuated in CuZnSOD transgenic mice [80], [117]. Subsequent studies have shown that DA-generated quinones, which trigger quinone cycling-dependent generation of superoxides and hydrogen peroxide, are indeed involved in METH toxicity [118], [119]. Studies measuring lipid peroxidation, activity of antioxidant enzymes, and formation of oxygen-based radicals have confirmed a role for free radicals in the mediation of METH toxicity [2], [82]. Our observations of significant increases in antioxidant transcripts suggest that repeated nontoxic oxidative stress induced by METH preconditioning might have triggered the development of a latent METH tolerance in striatal DA terminals whose cell bodies are located in midbrain DA neurons [120]. Moreover, the changes observed in antioxidant transcripts in SN/VTA cell bodies might serve to supply antioxidant proteins to scavenge METH-mediated DA-dependent reactive oxygen species generated within monoaminergic cell bodies and terminals [2], [80]. Thus, working jointly with BDNF, c-fos, and HspB2, increased transcription of these antioxidant genes might have promoted a state of resistance to any further METH-induced damage to the nigrostriatal DA system (see the schema in Fig. 8).

Also of interest are the increases in Hmox-1 expression observed after the METH challenge in the METH preconditioned group. Hmox-1 is a phase 2 enzyme that is induced by oxidative stress and cellular injury [121], [122]. METH causes its toxic effects, in part, by causing oxidative stress [80], [82]. The drug has recently been found to increase Hmox-1 expression [33], [83] and Hmox-1 induction protects against METH-induced toxicity [83]. Thus, METH preconditioning might be associated with modifications in the promoter of the Hmox-1 gene in such a way as to render it more responsive to the injection of METH doses that are known to cause oxidative stress-induced injuries in the brain [2], [79], with the increases in Hmox-1 expression playing a significant role in the protection against METH-induced DA depletion observed in the presence of METH preconditioning. This suggestion is supported by the reports that Hmox-1 is involved in the protection afforded by hyperbaric preconditioning of spinal cord neurons [123] and by isoflurane preconditioning against glucose deprivation [124]. It is also consistent with the observations that Hmox-1 overexpression protects against 1-methyl-4-phenylpyridinium-induced toxicity against dopaminergic neurons [125].

In summary, this is the first demonstration that prior repeated injections of nontoxic doses of METH, which cause protection against METH-induced striatal DA depletion in the rat, are also associated with differential transcriptional responses to toxic METH doses in the ventral midbrain of rats. These findings suggest that METH preconditioning protects against striatal DA depletion, in part, by suppressing injurious mechanisms while also augmenting neuroprotective pathways in the nigrostriatal dopaminergic pathway. Thus, the protective effects observed after METH preconditioning are not solely dependent on changes in one specific biochemical or molecular pathway but on multiple endogenous protective systems working in concert. Our observations further suggest that METH preconditioning-induced transcriptional alterations might be the results of epigenetic switches that affect promoter regions of genes in such a way that changes in their transcriptional regulation become manifest only in the presence of challenges with toxic doses of the psychostimulant. Finally, because our results are consistent with observations reported in models of brain preconditioning mediated by ischemia or pharmacological agents, it will be of interest to test if METH preconditioning might exert neuroprotective effects against other models of neurodegeneration.

## Materials and Methods

### Animals

Male Sprague-Dawley rats (Charles Rivers Laboratories, Raleigh, NC), weighing 330–370 g in the beginning of the experiment were used in the present study. Animals were housed in a humidity- and temperature-controlled room and were given free access to food and water. All animal procedures were performed according to the National Institutes of Health *Guide for the Care and Use of Laboratory Animals* and were approved by the local Animal Care Committee.

### Drug Treatment and Tissue Collection

Following habituation, rats were injected intraperitoneally with either (±)-METH-hydrochloride (NIDA, Baltimore, MD) or an equivalent volume of 0.9% saline for a period of three weeks as described in Table S1 in supplemental data. The saline- or METH-pretreated animals received either saline or METH (5 mg/kg×8 at 1 h intervals) challenges 72 hours after the preconditioning period. Similar doses of METH are known to cause significant decreases in the levels of monoamines in the rat striatum [12], [18] which received dopaminergic terminals from midbrain dopaminergic cell bodies located in the substantia nigra (SN) and ventral tegmental area (VTA) [126], [127]. Thus, the four groups of animals were: saline/saline (SS), saline/METH (SM), METH/saline (MS), and METH/METH (MM). The animals were euthanized 24 h later by decapitation. Their brains were quickly removed, striatal and SN/VTA tissues were dissected on ice, snap frozen on dry ice, and stored at −80°C until used in either HPLC, microarray analyses, or quantitative PCR experiments as described below. One side of the brain was used for HPLC and the other side for microarray and PCR experiments.

### HPLC

For monoamine analysis, the brain regions were homogenized in 0.01 M HClO_4_ and centrifuged at 14, 000×g for 15 min. DA, 3,4-dihydroxyphenylacetic acid (DOPAC), homovanillic acid (HVA), 5-HT and 5-hydroxyindoleacetic acid (5-HIAA) levels were analyzed in the brain tissue extracts using HPLC with electrochemical detector as previously described [128]. Monoamine levels are reported as pg/mg of tissue weight.

### RNA extraction

Total RNA was isolated using Qiagen RNeasy Midi kit (Qiagen, Valencia, CA) according to the manufacturer's instructions. RNA integrity was assessed using an Agilent 2100 Bioanalyzer (Agilent, Palo Alto, CA) and showed no degradation.

### Microarray hybridization and scanning

Microarray hybridization was carried out using Illumina's RatRef-12 Expression BeadChips arrays (22, 523 probes) (Illumina Inc., San Diego, CA). In brief, a 600 ng aliquot of total RNA from each striatal sample was amplified using Ambion's Illumina RNA Amplification kit (cat. no. IL1791; Ambion, Austin, TX). Single-stranded RNA (cRNA) was generated and labeled by incorporating biotin-16-UTP (Roche Diagnostics GmbH, Mannheim, Germany, cat. no. 11388908910). 750 ng of each cRNA sample were hybridized to Illumina arrays at 55°C overnight according to the Illumina Whole-Genome Gene Expression Protocol for BeadStation (Illumina Inc., San Diego, CA, cat. # 11201828). Hybridized biotinylated cRNA was detected with Cyanine3-streptavidin (Amersham Biosciences, Piscataway, NJ, cat. #146065) and quantified using Illumina's BeadStation 500GX Genetic Analysis Systems scanner as described previously [33].

### Microarray data analysis

The microarray data reported in the manuscript are in accordance with MIAME guidelines. The raw data for the analyses of the four groups of animals have been deposited in the NCBI GEO database: Accession number GSE17665. The Illumina BeadStudio software was used to measure fluorescent hybridization signals. Data were extracted by BeadStudio (Illumina, San Diego, CA) and then analyzed using GeneSpring software v. 7.3.1 (Silicon Genetics, Redwood City, CA, USA). Raw data were imported into GeneSpring and normalized using global normalization. The normalized data were used to identify changes in gene expression in these 3 group comparisons: MS vs SS, SM vs SS, and MM vs MS. A gene was identified as changed if it showed increased or decreased expression according to an arbitrary cut-off of 1.7-fold changes at p<0.05.

### Real-time PCR

Total RNA extracted from a midbrain region that encompasses the ventral tegmental area and substantia nigra of the rat was used to confirm the expression of genes of interest by real-time RT-PCR as previously described [33]. In brief, individual total RNA obtained from 5–7 rats per group was reverse-transcribed with oligo dT primers and RT for PCR kit (Clontech, Palo Alto, CA). PCR experiments were done using the Chroma4 RT-PCR Detection System (BioRad Hercules, CA USA) and iQ SYBR Green Supermix (BioRad) according to the manufacturer's protocol. Sequences for gene-specific primers corresponding to PCR targets were obtained using LightCycler Probe Design software (Roche). The primers were synthesized and HPLC-purified at the Synthesis and Sequencing Facility of Johns Hopkins University (Baltimore, MD). The list of primers is given in Table 4. Quantitative PCR values were normalized using 18S rRNA and quantified. The results are reported as relative changes which were calculated as the ratios of normalized gene expression data of each group compared to the SS group.

### Statistical Analysis

Statistical analysis was performed using analysis of variance (ANOVA) followed by Fisher's protected least significant difference post-hoc comparison (StatView 4.02, SAS Institute, Cary, NC). Values are shown as means ± SEM. The null hypothesis was rejected at p<0.05.

## Supporting Information

Table S1 (0.06 MB DOC)
