# Supplementary material for: Methamphetamine Preconditioning Alters Midbrain Transcriptional Responses to Methamphetamine-Induced Injury in the Rat Striatum
Source: PLoS One. 2009 Nov 12;4(11):e7812. doi: 10.1371/journal.pone.0007812 (PMC2771908; doi:10.1371/journal.pone.0007812)
Supplement: Table S1 — (0.06 MB DOC) [file pone.0007812.s001.doc]

Table S1. Schedule of METH pretreatment and challenges

|  | Monday | Tuesday | Wednesday | Thursday | Friday |
| --- | --- | --- | --- | --- | --- |
| Week 1 |  |  |  |  |  |
|  |  |  |  |  |  |
| 9:00 | 0.5 mg/kg | 1 mg/kg | 1 mg/kg | 1.5 mg/kg |  |
| 10:00 |  |  |  |  |  |
| 11:00 |  |  | 1 mg/kg | 1.5 mg/kg |  |
| 12:00 |  |  |  |  |  |
| 13:00 |  |  | 1 mg/kg | 1.5 mg/kg |  |
| 14:00 |  |  |  |  |  |
| 15:00 | 0.5 mg/kg | 1 mg/kg | 1 mg/kg | 1.5 mg/kg |  |
| 16:00 |  |  |  |  |  |
|  |  |  |  |  |  |
| Week 2 |  |  |  |  |  |
|  |  |  |  |  |  |
| 9:00 | 1 mg/kg | 1.5 mg/kg | 2 mg/kg | 2.5 mg/kg |  |
| 10:00 |  |  | 2 mg/kg | 2.5 mg/kg |  |
| 11:00 | 1 mg/kg | 1.5 mg/kg | 2 mg/kg | 2.5 mg/kg |  |
| 12:00 |  |  | 2 mg/kg | 2.5 mg/kg |  |
| 13:00 | 1 mg/kg | 1.5 mg/kg | 2 mg/kg | 2.5 mg/kg |  |
| 14:00 |  |  | 2 mg/kg | 2.5 mg/kg |  |
| 15:00 | 1 mg/kg | 1.5 mg/kg |  |  |  |
| 16:00 |  |  |  |  |  |
|  |  |  |  |  |  |
| Week 3 |  |  |  |  |  |
|  |  |  |  |  |  |
| 9:00 | 2 mg/kg |  | 5 mg/kg |  |  |
| 10:00 | 2 mg/kg |  | 5 mg/kg |  |  |
| 11:00 | 2 mg/kg |  | 5 mg/kg |  |  |
| 12:00 | 2 mg/kg |  | 5 mg/kg |  |  |
| 13:00 | 2 mg/kg |  | 5 mg/kg |  |  |
| 14:00 | 2 mg/kg |  | 5 mg/kg |  |  |
| 15:00 |  |  | 5 mg/kg |  |  |
| 16:00 |  |  | 5 mg/kg |  |  |

Initially the rats were divided into two groups, with one group receiving saline and the other group getting METH pretreatment according to the schedule described below during first and second weeks as well as Monday of the third week. The saline pretreatment was followed by with either saline (SAL/SAL) or METH challenges (SAL/METH), the METH pretreatment was followed by METH challenges (METH/METH) or METH followed by SAL(METH/SAL) on Wednesday of the third week and killed 24 h later.
